# Validation of the Charité Mobility assessment (CHARMI) in older adults across a wide range of functional levels - the prospective longitudinal MobiTest cohort study

**DOI:** 10.1007/s40520-026-03375-7

**Published:** 2026-03-28

**Authors:** Lea Kroboth, Filippo Maria Verri, Vanessa Haug, Tim Fleiner, Felix Böhm, Max E. Liebl, Michael Denkinger, Christoph Leinert

**Affiliations:** 1 Institute for Geriatric Research at AGAPLESION Bethesda Ulm, Ulm University Medical Center, Ulm, Germany; 2 Institute of Medical Engineering and Mechatronics, Ulm University of Applied Sciences, Ulm, Germany; 3 Department of Physical Medicine, Charité - Universitätsmedizin Berlin, Universität Berlin and Humboldt Universität zu Berlin, Berlin, Germany

**Keywords:** Mobility assessment, Geriatrics, CHARMI, Validation, Responsiveness, Minimal clinically important difference

## Abstract

**Background:**

Comprehensive mobility assessment is crucial in geriatrics, yet existing tools may lack feasibility, or applicability for older adults with varying functional levels.

**Aims:**

We evaluated the construct validity, interrater reliability, responsiveness, and minimal important change (MIC) of the Charité Mobility Index (CHARMI), in older adults.

**Methods:**

A prospective longitudinal cohort study was conducted with older adults ≥ 70y undergoing rehabilitation in acute care, inpatient or outpatient rehabilitation. CHARMI assessments were performed at admission and discharge and correlated with established mobility measures – the De Morton Mobility Index (DEMMI), Short Physical Performance Battery (SPPB), Timed Up and Go (TUG), and Barthel Index (BI) mobility items. Construct validity and responsiveness were evaluated using a hypothesis-testing approach based on a priori-hypotheses; interrater reliability (IRR) was assessed in a subsample. The MIC was determined in an anchor-based approach via ROC analysis.

**Results:**

93 participants (mean age 82.8y) were included. Evidence for validity was supported by meeting 7 of 8 a priori hypotheses including strong correlations with DEMMI and SPPB and a moderate inverse correlation with TUG. IRR was 0.97 (95% CI: 0,91; 0,99). Evidence for responsiveness was supported by meeting 6 of 8 a priori hypotheses. The MIC was established at 1.5 CHARMI points.

**Discussion and Conclusions:**

This study provides evidence supporting the CHARMI as a valid, responsive and reliable outcome measure with a useful MIC, making it a valuable option for tracking mobility in older adults across different settings and timepoints. Further studies should confirm generalizability and evaluate feasibility.

**Supplementary Information:**

The online version contains supplementary material available at 10.1007/s40520-026-03375-7.

## Introduction

To preserve functional independence, maintaining or improving mobility is a primary focus of geriatric rehabilitation and clinical care. For that purpose, accurate assessment of mobility is essential for identifying individuals at risk of functional decline and monitoring of therapeutic interventions. The broad spectrum of mobility impairments in older adults – from bedridden individuals to those capable of independent ambulation – requires tools that are both sensitive and applicable across this wide functional range [1].

However, even widely used and well-validated tools, such as the Timed Up and Go Test (TUG) [2], Short Physical Performance Battery (SPPB) [3], or the De Morton Mobility Index (DEMMI) [4] – have important limitations. The TUG assesses only a single timed transfer and walking task, which leads to substantial floor effects [5], especially in acute geriatric settings; in addition to that it has been described as insensitive to change at an individual patient level [6]. The SPPB, despite its robust validation in community and clinical populations, requires more materials (stop watch, 4-m walk, standardized chair) for balance, gait, and chair-rise components, and it too exhibits floor effects in severely impaired patients and ceiling effects in higher-functioning individuals [7]. The DEMMI is regarded as a gold-standard geriatric mobility measure, but it includes performance‑based tasks (e.g., transfers and ambulation) [4], that may limit feasibility within routine clinical workflows and in individuals who are unable to stand or walk. In light of this, Liebl et al. developed the Charité Mobility Index (CHARMI^®^) [8]. The CHARMI comprises 11 hierarchically ordered tasks spanning from bed mobility to full mobility (walking 1 km), is proxy-rated based on routine observations, and requires no special equipment. This design facilitates integration into daily care by physiotherapists or nursing staff and captures a broad spectrum of mobility, including severe impairment. Initial pilot use of CHARMI in mixed-age clinical populations indicated good feasibility and high clinical acceptance [8].

Despite its promising feasibility, the CHARMI has not yet undergone psychometric validation specifically in older aged cohorts. The original validation encompassed a wide age range (13–88 years) [8], leaving its measurement properties in geriatric populations insufficiently understood.

To address the clinical need for a feasible, equipment-free, and broadly applicable mobility assessment in geriatric care, our study aimed to evaluate the psychometric properties (validity, responsiveness, interrater reliability, and minimal important change - MIC) of the CHARMI in older adults across three clinical settings – acute geriatrics, inpatient rehabilitation, and outpatient rehabilitation – and to compare its performance with established mobility measures (DEMMI, SPPB, and TUG).

## Methods

### Study design and setting

This prospective longitudinal cohort study was conducted at Agaplesion Bethesda Hospital Ulm (Germany) between October 2023 and March 2024. Ethical approval was obtained by the ethical commission of Ulm University (#227/23).

### Participants

Patients aged 70 years or older admitted to acute geriatric care for early inpatient rehabilitation (approximately 8 therapy sessions per week over 3 weeks), inpatient geriatric rehabilitation (approximately 21 therapy sessions per week over 3 weeks), or outpatient geriatric rehabilitation (approximately 15 therapy sessions per week offered on 3 days per week over 5 weeks) were eligible for inclusion. All participants received routine care including a rehabilitative program depending on their setting. Exclusion criteria were patients suffering from advanced disease receiving palliative care (reduced life expectancy under three months by clinical judgement of treating physician), significant cognitive impairment (MMSE < 18 [9]), inability to understand the study information, severe communication barriers, or a diagnosis of delirium as documented in the electronic patient records.

### Recruitment strategy

Covering the wide spectrum of mobility levels among older adults, ranging from bedridden to fully mobile, has been a challenge in other studies, limiting the ability to assess floor and ceiling effects and covering only part of mobility levels [10]. To address a broader spectrum of mobility and considering recruitment feasibility, the target sample size was set at 10 participants per CHARMI category to achieve balanced representation of mobility levels. Since patients in acute geriatric care typically showed lower mobility levels compared to those in inpatient or outpatient rehabilitation, we prioritized recruitment in this setting. To minimize a potential source of selection bias, we aimed to approach all consecutive patients meeting the inclusion criteria across settings, inform them about the study, and invite them to participate.

### Mobility assessments

Mobility was evaluated at two time points (T1 and T2) during the participants’ stay in the three care settings: acute geriatric care with early inpatient rehabilitation, inpatient geriatric rehabilitation, and outpatient geriatric rehabilitation program. T1 assessments were conducted within four days of admission and T2 assessments 1–3 days before discharge, approximately 2–5 weeks after T1.

The four mobility assessments (CHARMI, DEMMI, SPPB, TUG) were applied at T1 and T2 in a fixed order to support feasibility and patient safety within routine clinical workflows. Baseline demographic and clinical information was obtained via structured patient interviews and review of electronic medical records. Mobility tests were administered by trained assessors via direct observation while participants performed standardized test tasks (with habitual walking aids if indicated). Tests requiring standing and walking (e.g., TUG) were attempted only when patients were able to mobilize, and assessments could be stopped if earlier tasks indicated that subsequent tests were not feasible, reducing unnecessary burden and potential fatigue. CHARMI was administered first and scored without reference to the results of the other mobility tests. CHARMI and SPPB are based on ordinal scales, while DEMMI uses an ordinal scale that is converted into an interval and TUG is measured on a ratio scale, as described below:

- Charité Mobility Index - CHARMI: Evaluates the performance in hierarchical key functional movements (e.g., bed mobility, transfers, walking) in older adults by observation. Scale: 0–10 (ordinal) [8].
- De Morton Mobility Index - DEMMI: A 15-item performance-based measure assessing mobility across various functional tasks (e.g., bed mobility, standing balance, walking). Scores range from 0 to 100, with higher scores indicating better mobility. Scale: 0–19 or 0–100 (ordinal or converted to interval) [4].
- Short Physical Performance Battery - SPPB: Assesses the performance of lower extremity function through three components: standing balance, gait speed, and the five-time chair stand test. Scale: 0–12 (ordinal) [3].
- Timed-up-and-go Test - TUG: Measures the time (in seconds) required to stand up from a chair, walk three meters, turn, return, and sit down, thereby assessing dynamic balance and gait speed. One timed trial was recorded and habitual walking aids were permitted. (ratio) [2];

All mobility tests were performed with each participant, guided by a trained physiotherapist, according to the respective test instructions and included the use of an aid (e.g. 4-wheeled walker, forearm walker, stick or forearm crutch) where appropriate and allowed in the test instructions.

### Data collection

In addition to the four mobility assessments, the following data was collected: baseline demographic and clinical data from electronic patient records, primary diagnoses, Barthel Index (BI) [9], Clinical Frailty Scale (CFS) [11], New Mobility Score (NMS) [12], Geriatric Depression Scale 5-item (GDS-5) [13], body mass index (BMI) [14], Visual Analogue Scale (VAS) for pain at rest [15], and Six-Item Screener (SIS) as a brief cognitive screening [16]. Even though, the BI was primarily intended to measure decline in institutional care of older adults [9], it is widely used as a relevant outcome measure also in acute care and rehabilitation settings [17, 18]. In addition to the BI total score (0–100), we derived two subscales a priori to split mobility‑related and non‑mobility activities of daily living (ADLs): a BI mobility subscore (sitting/standing/transfers; 0–40) and a BI non‑mobility subscore (eating, personal care, toileting/bathing/dressing, continence; 0–60). For descriptive comparability, we also expressed each subscore as a percentage of its subscale maximum (0–100%). These subscales were used for construct validity analyses (BI mobility for convergent validity; BI non‑mobility for divergent validity), while BI total was retained for anchor-based responsiveness and MIC analyses, because published and widely used MIC thresholds refer to the total Barthel Index score [17]. Nevertheless, we additionally examined responsiveness correlations between change in CHARMI and change in the BI mobility and BI non-mobility subscores to better reflect mobility-related change. Data collection was conducted with structured in-person interviews and assessments at T1 and T2.

Because participants were recruited across heterogeneous care settings and some had a certain degree of cognitive or communication limitations, we did not collect a patient-reported global rating of change. To assess the overall effect of the rehabilitative treatment on the participant in a global rating scale (GRS), an expert case review was conducted [19]. The review was performed by an experienced geriatrician (CL) using a 7-item-Likert scale including (1) ‘very much improved’, (2) ‘much improved’, (3) ‘a little improved’, (4) ‘no change’, (5) ‘a little deterioration’, (6) ‘much deterioration’ and (7) ‘very much deterioration’. Cases classified as (1) and (2) were defined as positive outcomes of the rehabilitative treatment. Cases classified (3) to (7) were defined as negative outcomes.

To minimize bias in data collection, the investigator (LK) responsible for data acquisition was trained by experienced physiotherapists and sports scientists, ensuring standardized measurement techniques. Additionally, interrater reliability was measured by reassessment of a convenience subsample of 17 participants by a second investigator (a trained physiotherapist), based on her availability, on the same day. All assessors involved in data collection were not involved in clinical care of the participants.

### Statistical analysis

Descriptive statistics summarized sample characteristics and outcomes. Continuous variables are presented as mean ± standard deviation (SD) or median with 25th-75th percentile, depending on distribution, and categorical data as counts and percentages.

We assessed the construct validity of the CHARMI – defined as the degree to which the instrument accurately measures the construct it intends to measure [20] – cross-sectionally at T1 by evaluating convergent and divergent validity. This involved establishing eight a priori hypotheses regarding the expected relationships between CHARMI and other relevant constructs. We employed Spearman’s rank correlations (ρ) to assess these relationships, reporting 95% confidence intervals. The strength of associations was interpreted using established thresholds: ρ ≤ 0.09 as negligible, 0.10 to 0.39 as weak, 0.4 to 0.69 as moderate, 0.70 to 0.89 as strong and ≥ 0.90 as very strong [21, 22]. To provide evidence for construct validity, we required confirmation of at least 75% (≥ 6/8) of the defined hypotheses [23].

Based on the construct coverage of the comparator instruments and previous psychometric work, we prespecified eight hypotheses for construct validity (Table 2). For convergent validity we hypothesized that: (1) CHARMI would demonstrate a strong to very strong positive correlation with DEMMI and (2) a strong to very strong positive correlation with SPPB (as all three instruments assess multi-component mobility across transfers, balance and ambulation [3, 4, 8]); (3) a moderate inverse correlation with TUG (reflecting its single timed transfer/walking task nature and potential floor effects in acutely impaired older adults [2, 5]); and (4) a moderate positive correlation with the BI mobility subscore, recognizing that this subscore is derived from an ADL index and was not designed as a stand-alone mobility test [9]. For divergent validity, we hypothesized that, (5) CHARMI would exhibit a weak to negligible correlation with the Six-Item Screener (SIS) for cognition; (6) a weak to negligible correlation with the GDS‑5 for mood; and (7) a weak to negligible correlation with a VAS for pain, as well as (8) a weaker positive correlation (≥ 0.2) with BI non-mobility items compared to BI mobility items, given their distinct construct relevance. Construct validity was considered sufficient if at least 75% (≥ 6/8) of the defined hypotheses were confirmed [23].

Analyses utilized complete‑case datasets; no imputation was performed, and sample sizes for each test are reported accordingly. Floor and ceiling effects were assessed for the DEMMI, SPPB, and TUG by calculating the proportion of participants who achieved the minimum or maximum scores, with > 15% indicating a substantial effect [23]. The CHARMI was not evaluated for floor or ceiling effects, due to the recruitment strategy aiming for a balanced distribution across all 11 CHARMI categories.

Building upon this framework for establishing construct validity, we extended the hypothesis-testing approach to evaluate the responsiveness of the CHARMI – defined as its ability to detect meaningful change over time [19]. This longitudinal construct validity assessment mirrors the methodology used for cross-sectional validity, differing primarily in the application of change scores. We formulated eight a priori hypotheses based on two types of comparisons: we employed 5 correlation coefficient tests between baseline and change scores and 3 anchor-based Receiver Operating Characteristic (ROC)/Area Under the Curve (AUC) thresholds. As with construct validity, we employed aforementioned thresholds for correlation strength and considered evidence for responsiveness sufficient if at least 75% (≥ 6/8) of hypotheses were confirmed [23].

(A) In analogy with the approach used for construct validity, for responsiveness testing with correlation coefficients we hypothesized that: ΔCHARMI would (1) demonstrate a strong positive correlation with ΔDEMMI; (2) at least a moderate positive correlation with ΔSPPB; (3) a weak and negative correlation with ΔTUG; and (4) a strong positive correlation with ∆BI mobility score, and (5) an at most moderately and positive correlation with ∆BI non-mobility score. These thresholds were prespecified based on conventional interpretations of correlation magnitudes and the expected degree of construct overlap, while acknowledging that correlations of change scores are typically attenuated by measurement error from repeated measurements [19, 20, 22]. Because TUG is only measurable in participants able to walk, shows substantial floor effects in early inpatient rehabilitation, and can be affected by changes in walking-aid use between time points, we expected weaker correlations for ΔTUG than for cross-sectional TUG.
(B) To further determine the responsiveness of the CHARMI with an anchor-based approach using ROC curves to discriminate between participants classified as improved versus not improved, as defined by three predefined external anchors, as described before [19, 24, 25]. For each anchor, participants were dichotomized using predefined thresholds: a MIC of the ≥ 10 points on the DEMMI [4, 26] – a well-established measure of mobility – and ≥ 11 points on the BI [17], a regularly used assessment of activities of daily living to measure overall rehabilitation success [27], as well as a positive rating from a global rehabilitation success rating scale (GRS) assessed by expert review. ΔCHARMI was treated as the test variable, and ROC curves were generated using ΔCHARMI cut-offs from 0.5 to 6.5 points in 1.0-point increments; sensitivity and specificity were computed at each threshold to derive the AUC (with 95% CI). AUC values were interpreted as follows: 0.90–1.00 (high accuracy), 0.70–0.89 (moderate), 0.50–0.69 (low), and < 0.50 (chance) [28], with an at least moderately accuracy ≥ 0.70 indicating a valuable model [19]. Based on this framework we added to the hypothesis testing for responsiveness that ΔCHARMI would discriminate between improved and not-improved patients for (6) at least moderately for DEMMI as an anchor, (7) at least moderately for BI as an anchor; and (8) at least moderately for GRS as an anchor.

Inter-rater reliability was assessed in a convenience subsample (*n* = 17) assessed independently on the same day by two trained assessors; inclusion was based on concurrent assessor availability. For continuous data (TUG), we used intraclass correlation coefficients (ICC) (2,1) with two-way random-effects and absolute agreement [29]. A weighted Cohen’s kappa coefficient was calculated for ordinal scales (CHARMI, DEMMI, SPPB) [29]. We considered a value of ICC [30] or weighted Cohen’s kappa of ≥ 0.7 as positive [31].

The minimal important change (MIC) [32] is defined as the smallest change in score in the construct to be measured that is perceived as important by patients, clinicians or relevant others, and would lead to a change in patient management [19, 33]. The MIC of the CHARMI was determined with an anchor-based approach by calculating the respective Youden indices of the AUCs of the different ROC with above mentioned clinical test (DEMMI, BI) – or expert-derived meaningful anchors (GRS) indicating the optimal threshold for maximizing the ability to separate participants that improved in a relevant way vs. not improved [19]. We selected the Youden index a priori as an objective summary cut-off that balances sensitivity and specificity; however, the clinical preference for minimizing false positives vs. false negatives may differ by use case. Anchor-specific MIC estimates were reported. If anchors produced different optimal cut-offs, we planned to derive a single summary MIC as the median of the anchor-specific thresholds.

Participants with interruption of the rehabilitation course due to transfer to another level of care (*n* = 4) were excluded from responsiveness and MIC analyses because follow-up assessments were not obtained under comparable discharge conditions. All tests were two‑sided with α = 0.05. All statistical analyses were performed using *SPSS* (version 29.0.1.0) and *R* (version 4.5.1) with *RStudio*, with relevant packages (*irr*,* psych*,* effsize*,* dplyr*,* pROC*). Statistical significance was defined as *p* < 0.05.

## Results

During the recruitment period from November 2023 until March 2024, a total of 978 patients were admitted to acute geriatric care, inpatient and outpatient rehabilitation and assessed for eligibility. After administration of the inclusion criteria and asking for willingness to participate, a total of 97 patients were recruited, including 51 patients from acute geriatrics, 23 from inpatient rehabilitation, and 23 from outpatient rehabilitation. Thirteen participants were lost to follow-up: four withdrew or were unable to complete baseline assessments, additional nine completed T1 but were discharged before T2. 93 participants were available for final analysis related to values at T1, whilst 84 were available at T2 that was conducted in median 17 days after T1. Baseline characteristics are shown in Table 1. The homogeneous distribution of the CHARMI scores of the enrolled participants at T1 was linked to the recruitment strategy and is shown together with the change of CHARMI from T1 to T2 in Supplementary Figure S1.

**Table 1:** Baseline characteristics. Characteristics of Study Participants

Demographic Characteristics (*n* = 93)		
Age (mean ± SD, Range)	82.80 ± 5.18 years, 71–94	
Sex (female, n [%])	64 (68.8%)	
Body Mass Index (BMI) (mean ± SD)	26.15 ± 4.84 kg/m^2^	
Living situation before admission n [%]) At Home Assisted living facility Nursing home	*n* = 78 (83.9%) *n* = 12 (12.9%) *n* = 3 (3.2%)	
Recruitment setting (n [%]) acute geriatrics incl. early inpatient rehabilitation inpatient rehabilitation outpatient rehabilitation	*n* = 49 (52.7%) *n* = 23 (24.7%) *n* = 21 (22.6%)	
Length of stay (mean ± SD)	26.18 ± 14.2 days	

Clinical Characteristics (*n* = 93)		
Primary diagnosis or reason for admission (n [%]) Cardiopulmonary Diseases Gait disturbances and Neurological Diseases Fractures Musculoskeletal Diseases Gastrointestinal Diseases Infections and Sepsis	*n* = 13 (14%) *n* = 27 (29%) *n* = 30 (32.3%) *n* = 6 (6.5%) *n* = 2 (2.1%) *n* = 15 (16.1%)	
Number of Secondary diagnosis (median (25–75))	10 (7–13.5)	
Clinical Frailty Scale (CFS) score 2 weeks prior (median (25–75))	4 (3–5)	
New Mobility Score (NMS) (median (25–75))	6 (5–8)	
Six-Item-Screener (SIS) (median (25–75))	6 (5–6)	
Geriatric Depression Scale 5-items (GDS 5) (median (25–75))	1 (0–1)	

Assessments (*n* = 93)		
Interval between Assessments T1 and T2 (median (25–75)) & range	17 (15–24.25) days range = 11–69 days	

	**T1 (n = 93)**	T2 (*N* = 84)
Charité Mobility Index (CHARMI) score (median (25–75))	5 (2.5–8)	8 (6.25–9)
De Morton Mobility Index (DEMMI) score (mean ± SD)	39.47 ± 18.74	48.15 ± 17.98
Short Physical Performance Battery (SPPB) score (median (25–75))	3 (0–6)	5 (2–7)
Timed up and Go time (TUG) time in seconds (median (25–75))	19.10 (13.22–27.10) (*n* = 48)*	21.55 (15.69–27.63) (*n* = 69)*
Barthel Index (BI) score at baseline (median (25–75))	55 (45–80)	75 (65–93.75)
Barthel Index Mobility score at baseline (median (25–75))	20 (10–30)	30 (20–35)
Barthel Index Non-Mobility score at baseline (median (25–75))	40 (30–50)	50 (40–55)
Visual analogue scale pain (VAS) in rest (median (25–75)) on exertion (median (25–75)))	2 (0–4) 4 (0–6)	0 (0–2) 1.5 (0–4)

Data are presented as mean ± standard deviation, median (25th-75th percentile) or absolute numbers (percentages), as appropriate; BMI = body mass index; SD = standard deviation. *deviating number of participants

### Validity and interrater reliability (IRR)

**Fig. 1:**
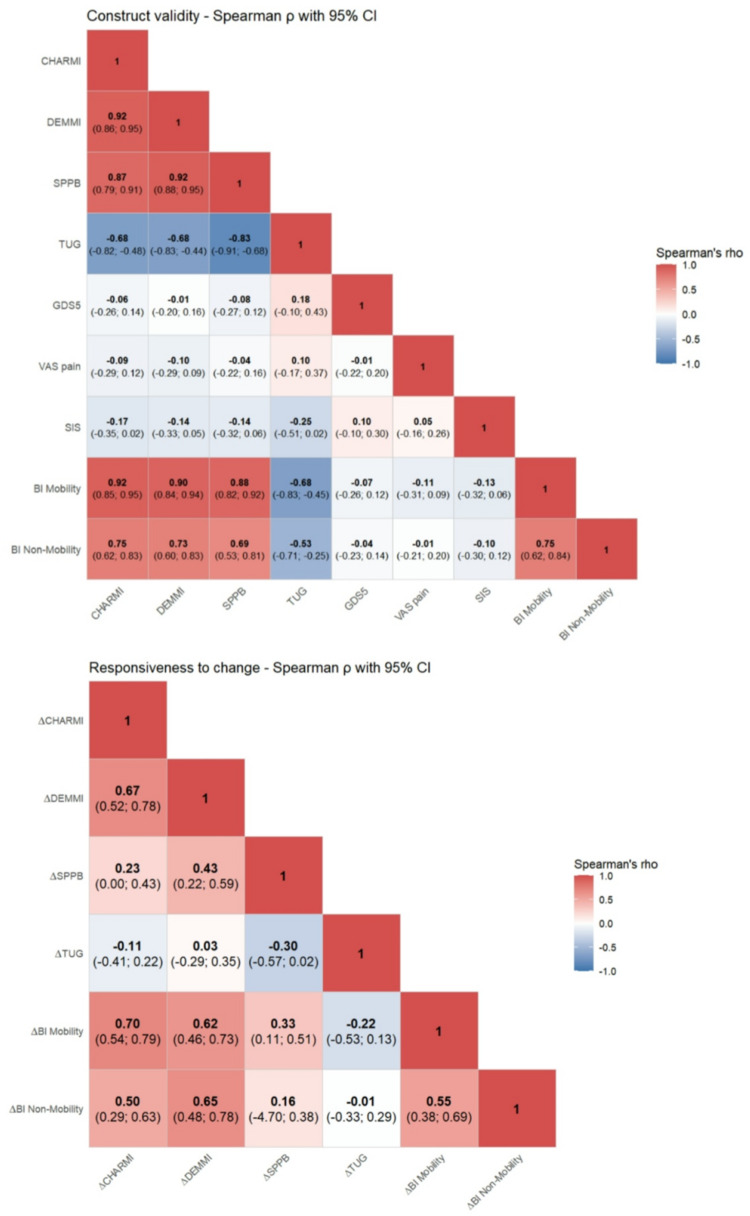
Correlation heatmaps of assessments. The heatmap displays Spearman’s correlation coefficient between various assessments, including mobility-related and other clinical measures. Color intensity indicates the strength and direction of the correlations (blue = negative, red = positive). Figure 1A) Validity: Assessments were collected at T1. Fig. 1B) Responsiveness: Change between T2-T1(∆) of CHARMI, DEMMI, SPPB, TUG and BI. *CHARMI = Charité Mobility Index; DEMMI = De Morton Mobility Index; SPPB = Short Physical Performance Battery; TUG = Timed Up and Go Test (in seconds*,* higher = worse); Barthel*,* BI = Barthel Index; BI Mobility % = mobility-related part (sitting/standing/transfers) in %; BI Non-Mobility % = non-mobility part (eating*,* personal care*,* toileting/bathing/dressing*,* continence in %); GDS-5 = Geriatric Depression Scale 5-items; VAS = Visual analogue scale*

Construct validity hypotheses (Table 2) were largely confirmed. Convergent validity showed very strong correlations between CHARMI and DEMMI (ρ = 0.92) and between CHARMI and SPPB (ρ = 0.87), and a moderate inverse correlation with TUG (ρ = -0.68). CHARMI also correlated very strongly with the BI mobility subscore (ρ = 0.92). Divergent validity hypotheses were supported by weak to negligible correlations with SIS (ρ = -0.17), GDS-5 (ρ = -0.06), and VAS pain (ρ = -0.09). The prespecified relative difference between correlations with BI mobility and BI non-mobility (ρ ≥ 0.20) was narrowly not met (ρ = 0.17). Overall, 7 of 8 construct validity hypotheses (87.5%) were confirmed (Fig. 1A; Supplementary Table S1).

**Table 2:** Ten a priori hypotheses for construct validity and to assess responsiveness of the CHARMI. Hypotheses formulated a priori to assess the responsiveness of the CHARMI

Hypotheses		Test result	Hypothesis met/unmet
Validity			
1	CHARMI is expected to correlate at least strongly and positively with DEMMI (ρ ≥ 0.7)	ρ = 0.92	+
2	CHARMI is expected to correlate at least strongly and positively with SPPB (ρ ≥ 0.7)	ρ = 0.87	+
3	CHARMI is expected to correlate moderately and negatively with TUG (ρ ≥ -0.4)	ρ = -0.68	+
4	CHARMI is expected to correlate moderately and positively with BI mobility items (ρ ≥ 0.4)	ρ = 0.92	+
5	CHARMI is expected to correlate at most weakly with SIS (ρ ≤ 0.4)	ρ = -0.17	+
6	CHARMI is expected to correlate at most weakly with GDS-5 (ρ ≤ 0.4)	ρ = -0.06	+
7	CHARMI is expected to correlate at least weakly with VAS (ρ ≤ 0.4)	ρ = -0.09	+
8	CHARMI is expected to correlate weaker and positively to BI non-mobility items compared to BI mobility items (≥ 0.2)	ρ = -0.17	-
**Total validity hypotheses met**			**7/8(87**,**5%)**

Responsiveness			
1	∆CHARMI is expected to correlate strongly and positively with ∆DEMMI (ρ ≥ 0.7)	ρ = 0.67	-
2	∆CHARMI is expected to correlate at least moderately and positively with ∆SPPB (ρ ≥ 0.4)	ρ = 0.23	-
3	∆CHARMI is expected to correlate weakly and negatively with ∆TUG (ρ ≤ -0.1)	ρ = -0.17	+
4	∆CHARMI is expected to correlate strongly and positively with ∆BI Mobility score (ρ ≥ 0.7)	ρ = 0.70	+
5	∆CHARMI is expected to correlate at most moderately and positively with ∆BI Non-Mobility score (ρ < 0.7)	ρ = 0.50	+
6	∆CHARMI is expected to identify “improved” patients based on DEMMI (≥ 10 according to [4, 26]) with an area under the ROC curve (AUC) ≥ 0.70 [19]	AUC 0.83	+
7	∆CHARMI is expected to identify “improved” patients based on BI (≥ 11according to [17]) with AUC ≥ 0.70	AUC 0.87	+
8	∆CHARMI is expected to identify “improved” patients based on expert review (GRS positive rehab effect) with AUC ≥ 0.70	AUC 0.81	+
**Total responsiveness hypotheses met**			**6/8 (75%)**

+: hypothesis met; - = hypothesis unmet

AUC – Area under the curve, BI – Barthel Index, CHARMI – Charité Mobility Index, ∆ = T2 – T1, DEMMI – De Morton Mobility Index, GDS-5 – Geriatric Depression Scale 5-item, GRS – Global Rating scale, ROC – receiver operating characteristic, SIS – Six-Item Screener, SPPB – Short Physical Performance Battery, TUG – Timed up and Go, VAS – Visual Analogue Scale

### Floor and ceiling effects

TUG exhibited significant floor effects, with 48.4% of participants unable to complete the test at T1 and 17.9% at T2. The SPPB showed a floor effect at T1 (25.8% of participants scoring the minimum) but not at T2 (8.3% of participants scoring the minimum). In contrast, no floor or ceiling effects above 15% were observed for the DEMMI at T1 or T2.

Interrater reliability was observed in 17 patients for the CHARMI, with a Cohen’s kappa of 0.97 (95% CI: 0.91; 0.99), for DEMMI with 0.99 (95% CI 0.99; 0.99), for SPPB of 0.85 (95% CI 0.59; 0.96) and for the TUG with an ICC of 0.93 (95% CI 0.77; 0.98). For TUG only 11 of 17 patients were assessed, due to the inability of 6 participants to perform the test.

### Responsiveness

For responsiveness analyses, ΔCHARMI correlated moderately with ΔDEMMI (ρ = 0.67) and weakly with ΔSPPB (ρ = 0.23) and showed a weak inverse correlation with ΔTUG (ρ = -0.17). As hypothesized, ΔCHARMI correlated strongly with change in BI mobility (ρ = 0.70) and at most moderately with change in BI non-mobility (ρ = 0.50). Figure 1B shows a heatmap of respective correlations.

**Fig. 2:**
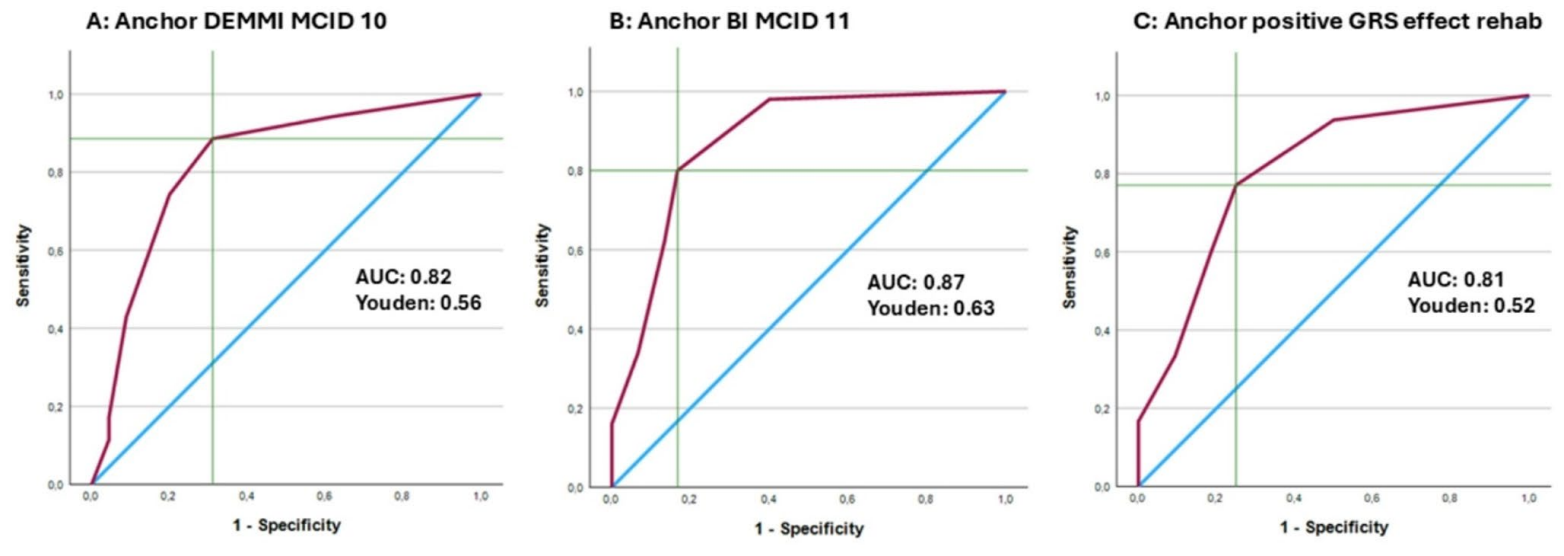
Receiver operating curves (ROC) of ∆CHARMI with different anchors. A: DEMMI MCID 10, B: BI MCID 11, C: GRS effect of rehabilitation positive

Anchor-based ROC analyses demonstrated good discrimination of improved versus not improved participants for all anchors (AUC 0.83 for DEMMI MIC, 0.87 for BI MIC, and 0.81 for expert GRS; Fig. 2; Supplementary Table S4). Overall, 6 of 8 hypotheses were confirmed, meeting the prespecified ≥ 75% criterion and supporting responsiveness of the CHARMI (Table 2).

Detailed analyses can be found in the Supplementary Table S2. Supplementary Figure S2 provides scatter plots for comparisons CHARMI vs. DEMMI, SPPB and TUG at T1 and T2 to facilitate visualization of relationship of the compared measures.

### Minimal important change (MIC)

We used three anchor-based methods to calculate the MIC of CHARMI. Based on the ROC curves of ∆CHARMI with the anchors DEMMI, BI and GRS rehab effect we found the Youden Index of each curve (Fig. 2). For DEMMI the Youden index 0.58 was found at sensitivity 0.89 and specificity of 0.69 corresponding with 1.5 ∆CHARMI points, for BI the Youden index 0.63 was found at sensitivity 0.8 and specificity 0.83 corresponding for 1.5 ∆CHARMI points and for GRS rehab effect the Youden index 0.52 was found at sensitivity 0.77 and specificity of 0.75 corresponding to 1.5 ∆CHARMI points. Consecutively the optimal combined median threshold could be found at ∆CHARMI 1.5 for all three anchors, identifying 1.5 points of the CHARMI as the MIC. For more detailed analyses, see the Supplementary Table S4.

## Discussion

This prospective cohort study across acute geriatric care, inpatient, and outpatient rehabilitation settings, provides evidence on psychometric properties of the CHARMI for mobility assessment in older adults and reports an anchor-based estimate of its MIC. Construct validity hypotheses were largely confirmed (7/8), showing high to very high convergent validity with DEMMI, SPPB and BI mobility and moderate inverse associations with TUG, while divergent validity was supported by weak correlations with SIS, GDS-5 and pain VAS and by a weaker association with BI non-mobility compared with BI mobility. Interrater reliability was high for CHARMI (κ = 0.97) and comparable to DEMMI (κ = 0.99), whereas SPPB showed lower agreement, and TUG could not be performed in a subset due to floor effects. Responsiveness hypotheses were met at the prespecified level (6/8), and anchor-based ROC analyses indicated that ΔCHARMI discriminated clinically meaningful improvement using established DEMMI and BI anchors and an expert GRS; across anchors, the optimal cut-off was 1.5 CHARMI points. We used the Youden index to select a balanced cut-off; depending on the intended clinical use, alternative thresholds emphasizing sensitivity or specificity could be considered.

Compared to the initial validation study [8] we found a stronger correlation of the CHARMI with the BI and an improved IRR, alongside a good responsiveness to change. In this first study, BI was used in total for discriminant validity with a correlation coefficient of 0.63, compared to 0.92 for BI total and 0.75 for the BI non-mobility items. The stronger correlations observed in our study may be attributed to the differing mean ages of the study populations (59 ± 16.3 years in the initial validation study and 82.80 ± 5.18 years in our study). The strong association between CHARMI and not only the mobility part of BI, but also the BI non‑mobility items, likely reflects the strong link between mobility capacity and the performance of all ADLs in older adults, particularly in rehabilitation settings where transfers and capacity to stand upright comprise the basis of many ADL tasks. Nevertheless, we consider the difference of 0.17 between correlation coefficients of CHARMI vs. BI mobility 0.92 and CHARMI vs. BI non-mobility 0.75 with non-overlapping CI as indicative for a relevant discriminative ability between motor and non-motor aspects of ADL. These patterns reinforce the construct specificity of CHARMI while acknowledging the expected interdependence of mobility and ADL domains in geriatric care.

Floor effects on admission (T1) were recognized for TUG with 48.4% and SPPB with 25.8%, but not for DEMMI. The predefined recruitment goals of 10 participants per CHARMI category prevented the analysis of ceiling and flooring effects for the CHARMI. Liebl et al. found a flooring effect of 20% on admission [8]. With the first mobility item of the CHARMI being “complete immobility”, we believe, that it would offer hardly relevant flooring effects even in acute care and early rehabilitation settings compared to SPPB and TUG.

The MIC of the CHARMI was determined by calculating the median of the Youden indices of the 3 anchors (DEMMI, BI, GRS). The combination of the 3 anchors broadened the perspective of functional “improved” outcome. The Youden index offers the best balance between false positive and false negative assessment results. In our context it is important to either reduce the risk for older adults that are estimated to have better mobility as they really do, but also not to underestimate mobility because of the risk to reduce treatment intensity or restrain from further rehabilitation measures. The calculated MIC of 1.5 points of the CHARMI leads to a practice-relevant change of 2 CHARMI categories given the ordinal 11-point scaling. In comparison, the MIC of DEMMI (100-point scale) in an acute care geriatric cohort has been described between 10 and 11 points [4, 26, 34, 35] leading practically to a change of 2 DEMMI categories on the 20-point scale and making the DEMMI slightly more sensitive than the CHARMI. Nevertheless, for the DEMMI, MICs between 6 and 14.65 points, depending on the setting and method [36, 37] have been reported. In a recent systematic review investigating the validity of SPPB, the minimal detectable change ranged from 0.7 to 3.42 depending on the cohort and method [7]. Only one study of the review calculated the MIC of the SPPB finding a MIC of 1.24 in a distribution and 2.25 in an anchor-based method [37]. For the TUG, the MIC was only reported in two studies in non-older aged cohorts of spinal surgery patients with a mean MIC of 3.4s [38] and 2.1s [39], that have a limited comparative value. Other assessments like the hierarchical assessment of balance and mobility (HABAM), that has been developed for the purpose of monitoring changes during acute care [40], was reported to be change sensitive; however, earlier evaluations used approaches that would not meet contemporary COSMIN standards for measurement property evaluation [20, 41].

Mobility is crucial to maintain independence in older age with strong prognostic value for disability and survival on a population level [1]. In emergency care, impaired mobility at presentation has greater prognostic value than vital signs for predicting 7-day mortality [42], underscoring its importance as an additional vital sign to be monitored during acute care [40, 43]. Beyond the acute and emergency care setting, mobility assessments also identify the need for rehabilitation as well as monitor and document the effects of interventions to improve physical activity [44] or rehabilitation progresses [45]. A mobility assessment should capture relevant changes in mobility in every and across all these settings and timepoints of a treatment course. Given its good validity and sensitivity to clinically relevant change in a heterogeneous older aged population, combined with its easy and fast clinical application, the CHARMI appears to be a promising assessment to fulfil this claim.

### Strength and limitations

A key strength of this study is that it is the first to test the CHARMI in a population of older adults, also calculating the MIC. The recruitment across multiple healthcare settings as well as the median Clinical Frailty Scale (CFS) score of 4 (3–5) enhances the representativeness and generalizability of the study sample for the often-heterogeneous older aged population.

A few limitations warrant consideration. Firstly, the single-center recruitment and modest sample size (*n* = 93), not reaching the initially planned 110 participants, due to difficulties in the recruitment of participants with poorer functional abilities in the given study period, may limit the generalizability of the findings. Nevertheless, our total sample (*n* = 93) exceeds commonly cited minimum thresholds (~ 50) for initial studies of measurement properties and psychometric testing [23]; however, it remains modest for subgroup analyses and for detecting small effects, so precision around some estimates is limited. Secondly, the pragmatic recruitment strategy of aiming for 10 participants per CHARMI category across three different clinical settings introduced a degree of recruitment bias and precluded certain statistical analyses that would have afforded a more unbiased distribution of participants. The recruitment of 97 patients (93 participants, 4 dropouts) out of 978 may, in this context, initially appear to be limited; however, this was primarily determined by the recruitment capacity of the study personnel and the planned stratification by CHARMI category, rather than a lack of suitability or willingness to participate. In addition, because no screening log was kept, we could not quantify the response rate or reasons for nonparticipation. Nonetheless, this strategy facilitated the time-efficient recruitment of a wide range of mobility levels within limited resources. Given ordinal scaling and non-normal distributions, we used non-parametric and hypothesis-testing approaches, complete-case analyses per outcome, and confidence intervals to support transparent interpretation. Thirdly, when determining the MIC we focused on score- and expert-based anchors and did not include a patient reported outcome measure (PROM). What patients identify as an important difference in mobility might depend on a variety of factors: e.g. the baseline mobility before an acute care incident that led to the deterioration as well as the current status of mobility before the rehabilitation measure, especially in our heterogenous cohort; their expectations on the planned rehabilitation measure and the success of previous rehabilitation treatments; patient-physician interactions; and external (e.g. another acute care problem) as well as intrinsic factors (e.g. mood of the patient) that might influence perception of the rehabilitation course [33, 46]. Nevertheless, we recommend including PROM in future evaluations in other more homogenous cohort. Fourthly, the exclusion of patients with severe cognitive impairment, delirium, or diagnosed dementia restricts the applicability of the CHARMI to these common subgroups of older adults, that might be included in further evaluations as well. Finally, while the fixed test sequence could have introduced fatigue effects, prioritizing the CHARMI as the initial assessment was essential to mitigate bias from knowledge of other assessment scores.

## Conclusion

In a prospective cohort of older adults across acute geriatric care and rehabilitation settings, the CHARMI showed evidence of good construct validity and responsiveness to change, an excellent IRR, and a MIC determined at 1.5 points in a diverse population of older adults in acute care, inpatient and outpatient rehabilitation. It is possibly a valuable option for tracking mobility changes in older adults across diverse geriatric care settings. In a next step, we will evaluate the equating and comparability of these mobility assessments to allow for standardized comparisons in various settings and studies. Future studies could encompass a Rasch analysis to assess item fit and Differential Item Functioning (DIF), as well as validation studies in bigger unselected multicenter samples to confirm generalizability. Furthermore, limited cross-sectional discrimination underscores the need for further refinement and validation, particularly in cognitively impaired populations and larger, multicenter cohorts.

## Supplementary Material

Below is the link to the electronic supplementary material.

Supplementary Material 1

## Data Availability

Data can be made available upon request.
