# Supplementary material for: Validation of the Charité Mobility assessment (CHARMI) in older adults across a wide range of functional levels - the prospective longitudinal MobiTest cohort study
Source: Aging Clin Exp Res. 2026 Mar 28;38(1):111. doi: 10.1007/s40520-026-03375-7 (PMC13065600; doi:10.1007/s40520-026-03375-7)
Supplement: Supplementary file 1 — Supplementary Material 1 [file 40520_2026_3375_MOESM1_ESM.docx]

**Supplementary Materials:**


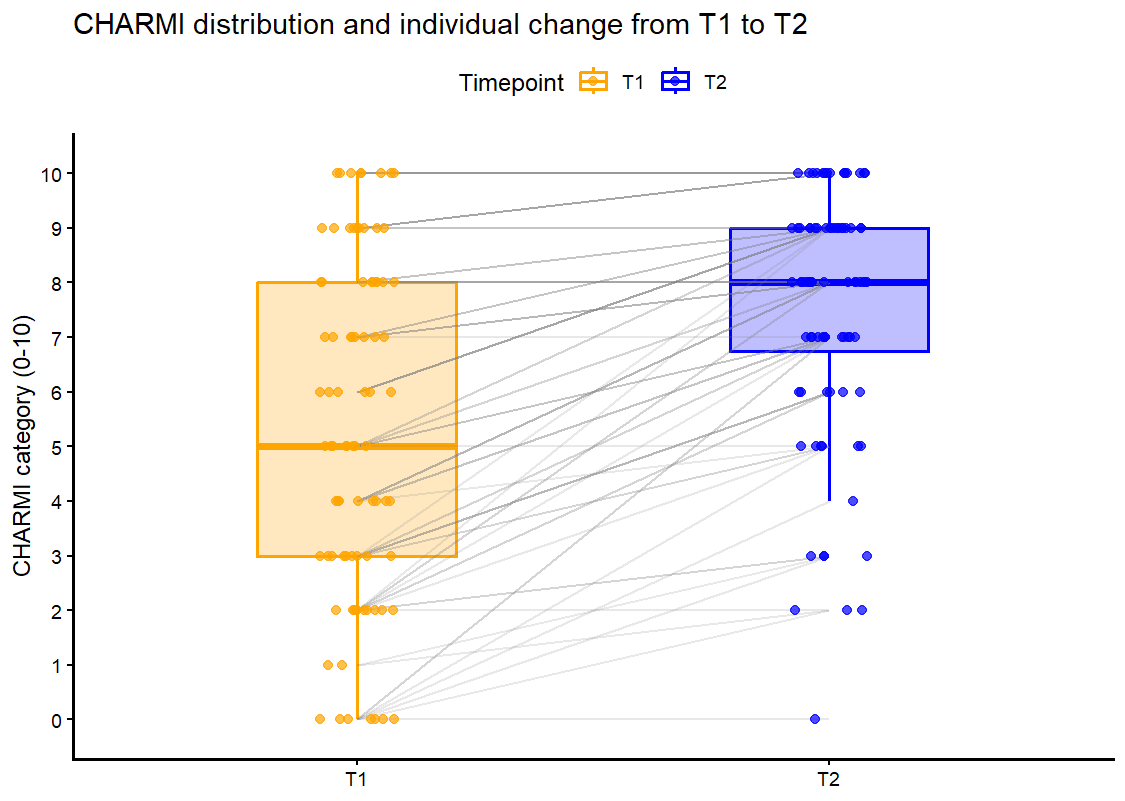


**Fig. S1: Scatterplot of the distribution of individual CHARMI scores at T1 (A) and T2 (B), with boxplots indicating the median and 25-75 IQR.** CHARMI = Charité Mobility Index


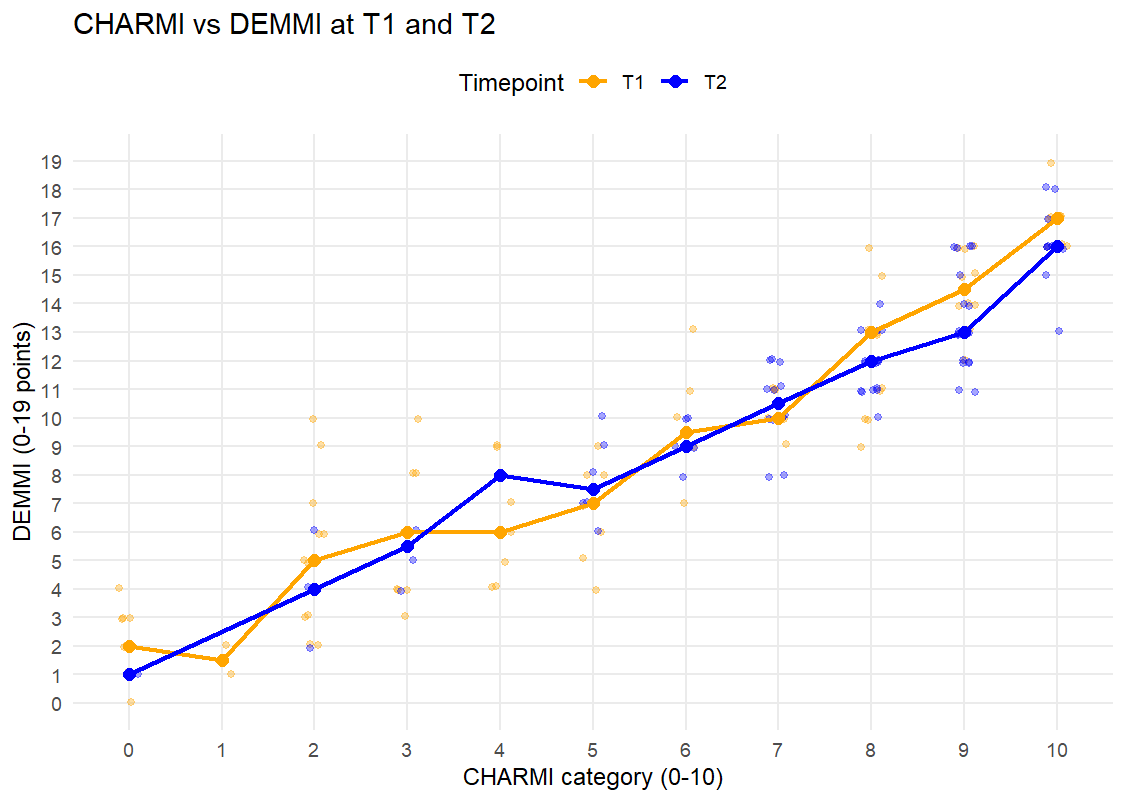

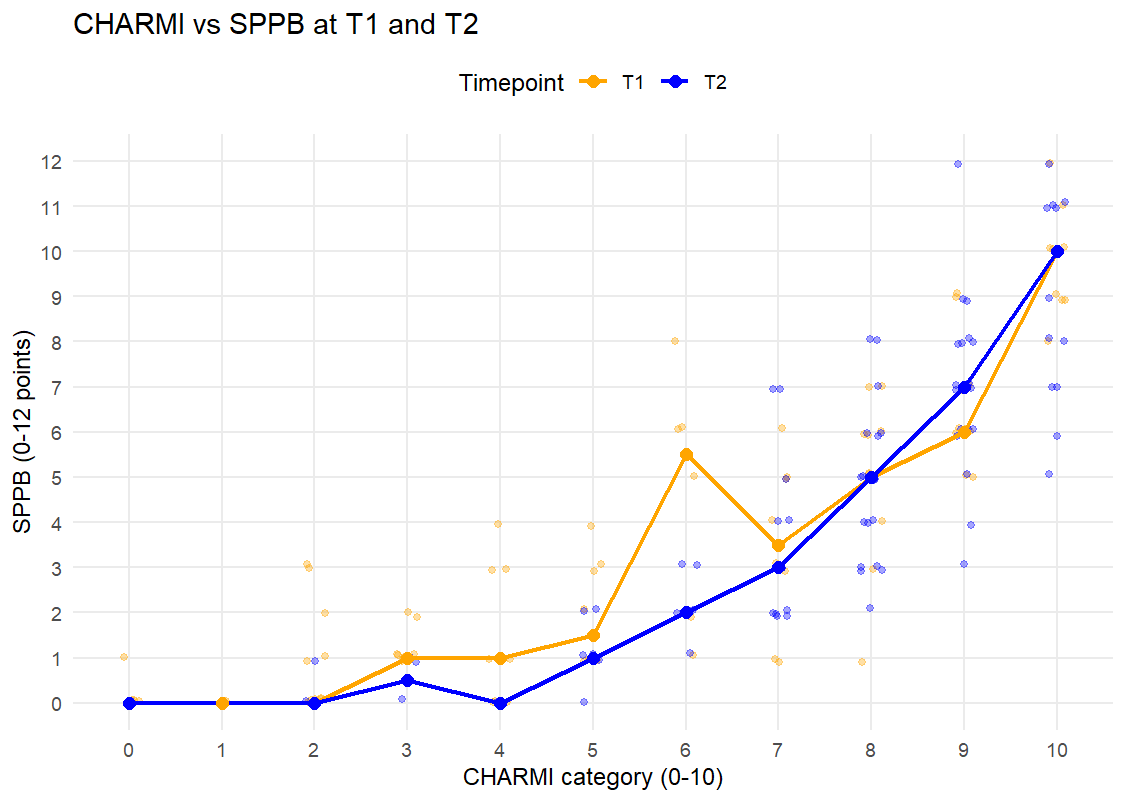

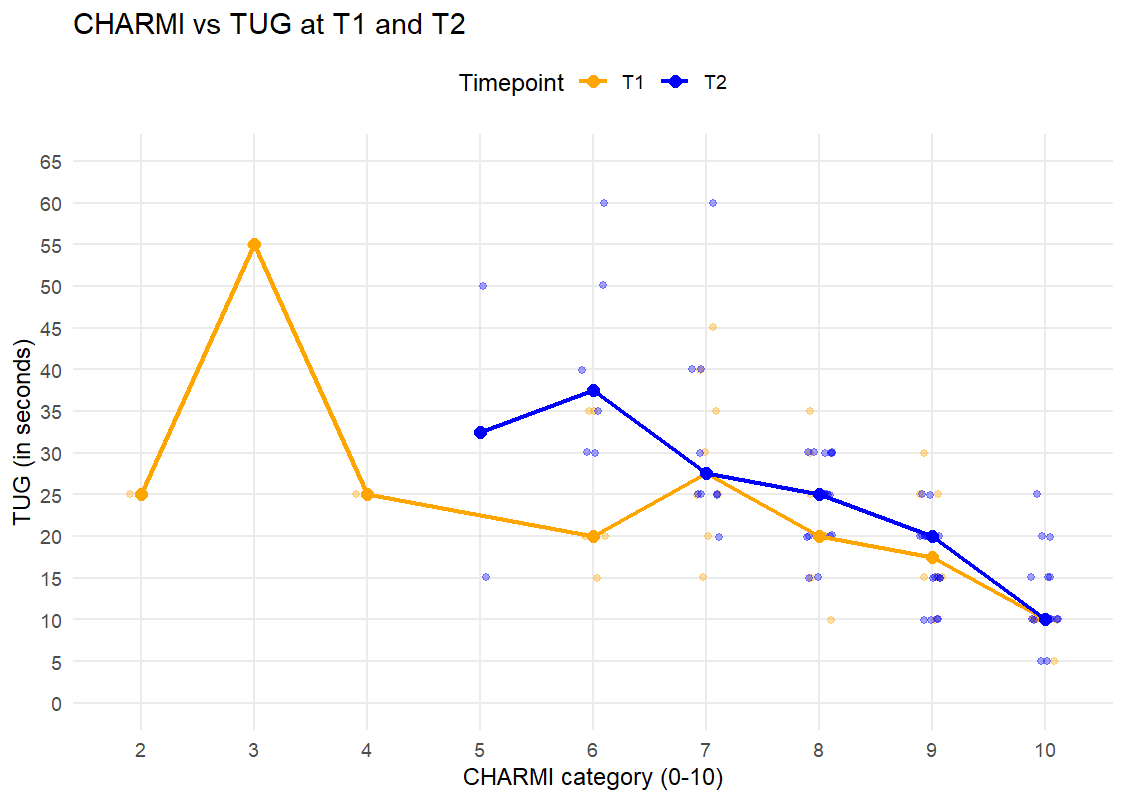


**Fig. S2: (a-c). Scatterplot showing the relationship between CHARMI (0-10) and three mobility measures at baseline (T1) and discharge (T2)**(a) CHARMI vs. DEMMI (0-19 points). (b) CHARMI vs. SPPB (0-12 points). (c) CHARMI vs. TUG (displayed in 5‑second steps).
CHARMI = Charité Mobility Index
DEMMI = De Morton Mobility Index
SPPB = Short Physical Performance Battery
TUG = Timed-up-and-go Test

| **Supplementary Table S1**. Spearman’s correlations between assessments at T1 | | | | | | |
| --- | --- | --- | --- | --- | --- | --- |
|  | | n | Spearman Rho | Significance (2-tailed) | Confidence intervals (two-sided)^a,b^ | |
|  |  |  |  |  |  |  |
|  |  |  |  |  |  |  |
|  |  |  |  |  | Lower | Upper |
| CHARMI | DEMMI | 93 | 0,917 | 5,10E-38 | 0,862 | 0,949 |
| CHARMI | Barthel Index (mobility) | 93 | 0,915 | 1,18E-37 | 0,852 | 0,954 |
| CHARMI | SPPB | 93 | 0,866 | 4,09E-29 | 0,793 | 0,913 |
| CHARMI | Barthel Index (non-mobility) | 93 | 0,746 | 9,27E-18 | 0,62 | 0,834 |
| CHARMI | TUG | 48 | -0,681 | 1,02E-07 | -0,815 | -0,48 |
| CHARMI | SIS | 93 | -0,167 | 1,10E-01 | -0,346 | 0,022 |
| CHARMI | VAS pain | 93 | -0,088 | 4,04E-01 | -0,294 | 0,124 |
| CHARMI | GDS-5 | 93 | -0,059 | 5,77E-01 | -0,26 | 0,141 |
| DEMMI | SPPB | 93 | 0,921 | 4,26E-39 | 0,876 | 0,949 |
| DEMMI | Barthel Index (mobility) | 93 | 0,902 | 6,52E-35 | 0,841 | 0,939 |
| DEMMI | Barthel Index (non-mobility) | 93 | 0,734 | 6,13E-17 | 0,598 | 0,833 |
| DEMMI | TUG | 48 | -0,675 | 1,46E-07 | -0,825 | -0,44 |
| DEMMI | SIS | 93 | -0,144 | 1,70E-01 | -0,329 | 0,05 |
| DEMMI | VAS pain | 93 | -0,098 | 3,52E-01 | -0,286 | 0,089 |
| DEMMI | GDS-5 | 93 | -0,012 | 9,08E-01 | -0,2 | 0,16 |
| SPPB | Barthel Index (mobility) | 93 | 0,878 | 6,62E-31 | 0,818 | 0,921 |
| SPPB | TUG | 48 | -0,83 | 3,07E-13 | -0,912 | -0,681 |
| SPPB | Barthel Index (non-mobility) | 93 | 0,688 | 2,55E-14 | 0,533 | 0,805 |
| SPPB | SIS | 93 | -0,136 | 1,94E-01 | -0,322 | 0,06 |
| SPPB | GDS-5 | 93 | -0,076 | 4,71E-01 | -0,271 | 0,117 |
| SPPB | VAS pain | 93 | -0,038 | 7,18E-01 | -0,219 | 0,16 |
| TUG | Barthel Index (mobility) | 48 | -0,675 | 1,40E-07 | -0,825 | -0,449 |
| TUG | Barthel Index (non-mobility) | 48 | -0,527 | 1,18E-04 | -0,708 | -0,254 |
| TUG | SIS | 48 | -0,253 | 8,33E-02 | -0,511 | 0,024 |
| TUG | GDS-5 | 48 | 0,176 | 2,31E-01 | -0,101 | 0,429 |
| TUG | VAS pain | 48 | 0,104 | 4,83E-01 | -0,169 | 0,37 |
| GDS-5 | Barthel Index (mobility) | 93 | -0,07 | 5,03E-01 | -0,262 | 0,124 |
| GDS-5 | Barthel Index (non-mobility) | 93 | -0,038 | 7,20E-01 | -0,232 | 0,137 |
| GDS-5 | VAS pain | 93 | -0,014 | 8,96E-01 | -0,216 | 0,202 |
| VAS pain | Barthel Index (mobility) | 93 | -0,109 | 2,99E-01 | -0,305 | 0,093 |
| VAS pain | Barthel Index (non-mobility) | 93 | -0,009 | 9,34E-01 | -0,205 | 0,196 |
| SIS | Barthel Index (mobility) | 93 | -0,132 | 2,06E-01 | -0,324 | 0,06 |
| SIS | GDS-5 | 93 | 0,097 | 3,55E-01 | -0,104 | 0,299 |
| SIS | Barthel Index (non-mobility) | 93 | -0,095 | 3,67E-01 | -0,299 | 0,117 |
| SIS | VAS pain | 93 | 0,052 | 6,19E-01 | -0,159 | 0,255 |
|  | a. Estimates are based on Fisher’s r-to-z transformation. | | | | | |
|  | b. The estimation of the standard error is based on the formula proposed by Fieller, Hartley, and Pearson. | | | | | |
| The table presents Spearman’s correlation coefficients (ρ) with sample size (n), 95% two-sided confidence intervals, and p-values between various assessments, including mobility related and other clinical measures. Assessments were collected at T1. | | | | | | |
| *CHARMI = Charité Mobility Index; DEMMI = De Morton Mobility Index; SPPB = Short Physical Performance Battery; TUG = Timed Up and Go Test; Barthel = Barthel Index; GDS-5 = Geriatric Depression Scale 5-items; VAS = Visual analogue scale* | | | | | | |

| **Supplementary Table S2**. Spearman’s correlations between change scores (∆) | | | | | | |
| --- | --- | --- | --- | --- | --- | --- |
|  | | N | Spearman-Rho | Significance(2-tailed) | 95% confidence intervals  (two-sided)^a,b^ | |
|  |  |  |  |  | Lower | Upper |
| ∆CHARMI - ∆DEMMI | | 80 | 0,671 | <,001 | 0,524 | 0,779 |
| ∆CHARMI - ∆SPPB | | 80 | 0,226 | 0,044 | 0 | 0,43 |
| ∆CHARMI - ∆TUG | | 40 | -0,107 | 0,512 | -0,413 | 0,221 |
| ∆CHARMI - ∆Barthel Index | | 80 | 0,667 | <,001 | 0,519 | 0,776 |
| ∆CHARMI - ∆BI Mobility | | 80 | 0,701 | <,001 | 0,54 | 0,793 |
| ∆CHARMI - ∆BI Non-Mobility | | 80 | 0,502 | <,001 | 0,29 | 0,629 |
| ∆DEMMI - ∆SPPB | | 80 | 0,425 | <,001 | 0,221 | 0,594 |
| ∆DEMMI - ∆TUG | | 40 | 0,034 | 0,835 | -0,289 | 0,35 |
| ∆DEMMI - ∆Barthel Index | | 80 | 0,652 | <,001 | 0,5 | 0,765 |
| ∆DEMMI - ∆BI Mobility | | 80 | 0,619 | <,001 | 0,46 | 0,73 |
| ∆DEMMI - ∆BI Non-Mobility | | 80 | 0,653 | <,001 | 0,482 | 0,777 |
| ∆SPPB - ∆TUG | | 40 | -0,302 | 0,059 | -0,567 | 0,02 |
| ∆SPPB - ∆Barthel Index | | 80 | 0,302 | 0,007 | 0,081 | 0,494 |
| ∆SPPB - ∆BI Mobility | | 80 | 0,33 | 0,003 | 0,113 | 0,505 |
| ∆SPPB - ∆BI Non-Mobility | | 80 | 0,159 | 0,159 | -0,047 | 0,383 |
| ∆TUG - ∆Barthel Index | | 40 | -0,126 | 0,437 | -0,429 | 0,202 |
| ∆TUG - ∆BI Mobility | | 40 | -0,223 | 0,167 | -0,534 | 0,127 |
| ∆TUG - ∆BI Non-Mobility | | 40 | -0,005 | 0,976 | -0,333 | 0,29 |
| ∆Mobility - ∆BI Non-Mobility | | 80 | 0,554 | <,001 | 0,381 | 0,69 |
|  | a. Estimates are based on Fisher’s r-to-z transformation. | | | | | |
|  | b. The estimation of the standard error is based on the formula proposed by Fieller, Hartley, and Pearson. | | | | | |
| The table presents Spearman’s correlation coefficients (ρ) with sample size (n), 95% two-sided confidence intervals, and p-values between assessments. Responsiveness is represented by change scores (∆) between T1 and T2 for CHARMI, DEMMI, SPPB, TUG and Barthel Index.  *CHARMI = Charité Mobility Index; DEMMI = De Morton Mobility Index; SPPB = Short Physical Performance Battery; TUG = Timed Up and Go Test; Barthel = Barthel Index; BI Mobility = Mobility elements of Barthel Index; BI Non-Mobility = Non-Mobility elements of Barthel Index.* | | | | | | |

| **Supplementary Table S3**. Comparison of ΔCHARMI scores between improved and not improved participants across anchors | | | | | | | |
| --- | --- | --- | --- | --- | --- | --- | --- |
|  |  | Anchor DEMMI ≥ 10 | | Anchor Barthel ≥ 11 | | Anchor GRS positive rehab effect | |
|  |  | Improved | Not-Improved | Improved | Not-Improved | Improved | Not-Improved |
| N | | 35 | 45 | 50 | 30 | 48 | 32 |
| median (25-75) | | 3 (2-4) | 1 (0-2) | 3 (2-4) | 0 (0-1) | 3 (2-4) | 0.5 (0-1.75) |
| Mann-Whitney U test | Hypothesis | The distribution of ΔCHARMI is the same across categories of Anchor DEMMI≥10 | | The distribution of ΔCHARMI is the same across categories of Anchor Barthel≥11 | | The distribution of ΔCHARMI is the same across categories of Anchor GRS effect rehab | |
|  | U statistic | 1296 | | 1311 | | 1241 | |
|  | p-value | <0.001 | | <0.001 | | <0.001 | |
| The table presents the comparison of ΔCHARMI scores (median and 25^th^-75^th^ percentiles) between categories of improvement according to three anchors: DEMMI (MIC ≥ 10), Barthel Index (MIC ≥ 11), and expert review (global rating scale of rehabilitation effect). Sample sizes (n) are reported for each group. Group differences were tested using Mann-Whitney U tests for two independent groups, with the U statistics and asymptotic two-tailed p-values provided. The null hypotheses tested whether the distribution of ΔCHARMI is the same across improvement categories for each anchor. | | | | | | | |

| **Supplementary Table S4.** ROC metrics of ΔCHARMI scores between improved and not improved participants across anchors | | | |
| --- | --- | --- | --- |
| Area under the ROC curve of ΔCHARMI scores with anchor DEMMI ≥ 10 | | | |
| Area | Std. Error (a) | Asymptomatic 95% |  |
|  |  | Lower Bound | Upper Bound |
| 0,82 | 0,05 | 0,73 | 0,92 |
| Coordinates of the ROC curve of ΔCHARMI scores with anchor DEMMI ≥ 10 | | | |
| Positive if Greater Than or Equal To (b) | Sensitivity | 1 - Specificity | Youden's Index |
| -1 | 1 | 1 | 0 |
| 0,50 | 0,94 | 0,62 | 0,32 |
| 1,50 | 0,89 | 0,31 | 0,58 |
| 2,50 | 0,74 | 0,20 | 0,54 |
| 3,50 | 0,43 | 0,09 | 0,34 |
| 4,50 | 0,17 | 0,04 | 0,13 |
| 5,50 | 0,11 | 0,04 | 0,07 |
| 6,50 | 0,06 | 0,02 | 0,04 |
| 8 | 0 | 0 | 0 |
| Area under the ROC curve of ΔCHARMI scores with anchor Barthel Index ≥ 11 | | | |
| Area | Std. Error (a) | Asymptomatic 95% |  |
|  |  | Lower Bound | Upper Bound |
| 0,87 | 0,04 | 0,79 | 0,96 |
| Coordinates of the ROC curve of ΔCHARMI scores with anchor Barthel Index ≥ 11 | | | |
| Positive if Greater Than or Equal To (b) | Sensitivity | 1 - Specificity | Youden's Index |
| -1 | 1 | 1 | 0 |
| 0,50 | 0,98 | 0,40 | 0,58 |
| 1,50 | 0,80 | 0,17 | 0,63 |
| 2,50 | 0,62 | 0,13 | 0,49 |
| 3,50 | 0,34 | 0,07 | 0,27 |
| 4,50 | 0,16 | 0 | 0,16 |
| 5,50 | 0,12 | 0 | 0,12 |
| 6,50 | 0,06 | 0 | 0,06 |
| 8 | 0 | 0 | 0 |
| Area under the ROC curve of ΔCHARMI scores with anchor GRS effect rehab | | | |
| Area | Std. Error (a) | Asymptomatic 95% |  |
|  |  | Lower Bound | Upper Bound |
| 0,81 | 0,05 | 0,71 | 0,91 |
| Coordinates of the ROC curve of ΔCHARMI scores with anchor GRS effect rehab | | | |
| Positive if Greater Than or Equal To (b) | Sensitivity | 1 - Specificity | Youden's Index |
| -1 | 1 | 1 | 0 |
| 0,50 | 0,94 | 0,50 | 0,44 |
| 1,50 | 0,77 | 0,25 | 0,52 |
| 2,50 | 0,60 | 0,19 | 0,42 |
| 3,50 | 0,33 | 0,09 | 0,24 |
| 4,50 | 0,17 | 0 | 0,17 |
| 5,50 | 0,13 | 0 | 0,13 |
| 6,50 | 0,06 | 0 | 0,06 |
| 8 | 0 | 0 | 0 |
| a. Under the nonparametric assumption b. The smallest cutoff value is the minimum observed test value minus 1, and the largest cutoff value is the maximum observed test value plus 1. All the other cutoff values are the averages of two consecutive ordered observed test values. | | | |
